# The role of pyroptosis in modulating the tumor immune microenvironment

**DOI:** 10.1186/s40364-022-00391-3

**Published:** 2022-06-23

**Authors:** Jinxiang Wu, Lei Wang, Jianwei Xu

**Affiliations:** 1 Department of Pulmonary and Critical Care Medicine, Qilu Hospital, Cheeloo College of Medicine, Shandong University, Jinan, China; 2 Department of Pancreatic Surgery, General Surgery, Qilu Hospital, Cheeloo College of Medicine, Shandong University, Jinan, 250012 Shandong Province China

**Keywords:** Pyroptosis, Tumor immune microenvironment, Gasdermin, Immune checkpoint

## Abstract

The tumor immune microenvironment (TIME) plays a key role in immunosuppression in cancer, which results in tumorigenesis and tumor progression, and contributes to insensitivity to chemotherapy and immunotherapy. Understanding the mechanism of TIME formation is critical for overcoming cancer. Pyroptosis exerts a dual role in modulating the TIME. In this review, we summarize the regulatory mechanisms of pyroptosis in modulating the TIME and the potential application of targeted pyroptosis therapy in the clinic. Several treatments targeting pyroptosis have been developed; however, the majority of treatments are still in preclinical studies. Only a few agents have been used in clinic, but the outcomes are unsatisfactory. More studies are necessary to determine the role of pyroptosis in cancer, and more research is required to realize the application of treatments targeting pyroptosis in the clinic.

## Overview of cancer burden

Cancer is a major global challenge. In 2019, respiratory cancer ranked as the sixth leading cause of death in the world [1]. In China, respiratory cancer was the third leading cause of death after stroke and ischemic heart disease, and liver cancer was the fifth leading cause in 2017 [2]. According to the estimate from Global Cancer Observatory, there will be approximately 4,820,000 and 2,370,000 new cancer cases and 3,210,000 and 640,000 cancer deaths in China and the USA in 2022, respectively [3]. Many cancer treatments have been developed; however, no tumor can be radically cured. For example, pancreatic cancer, the most malignant tumor, has a five-year survival of less than 10% [4]. The low rate of early detection, high incidence of recurrence after resection, and resistance to chemotherapy partly account for the poor prognosis of pancreatic cancer patients. To improve the prognosis of pancreatic cancer, novel therapeutic regimens have been developed, such as immunotherapy and targeted therapy. However, the outcomes of treatments, especially immunotherapy, have not met expectations [5]. The complex mechanisms of tumor development may partly account for the poor prognosis. The tumor immune microenvironment (TIME) plays a vital role in tumorigenesis and tumor progression, as well as to tumor sensitivity to chemotherapy and immunotherapy. Better understanding of the TIME may lead to therapeutic strategies for cancer treatment.

## The TIME

The TIME consists of cellular and acellular components, including cancer cells, tumor-associated stomal cells, immune cells, extracellular matrix, and cytokines and other soluble molecules. (Table 1). The processes and mechanisms of TIME formation are extremely complex and vary among tumors depending on the genetic background and heterogeneity. The TIME of pancreatic cancer is representative of its characteristics of fewer tumor cells (accounting for 10% of total cells), more infiltration of immunosuppressive cells and fewer effector T cells, extremely dense matrix, and immunosuppression [6–8]. Therefore, in this review, we focus on pancreatic cancer as an example to describe the complex networks of the TIME.

**Table 1:** The cellular components of the TIME in pancreatic cancer

Cells	Cell-derived cytokines	Function
Cancer cells	CCL-2, TGF-β, ICAM-1, GM-CSF, G-CSF, IL-10, IL-6, IL-1β	TAM (↑), MDSC (↑), PSC (↑), Treg (↑) CD8^+^T cells (↓), NK cells (↓)
aPSC/CAF	CCL3, CCL6, IL-6, CXCL1, CXCL12	Cancer cells (↑), TAM (↑), MDSC (↑) NK cells (↓), CD8 + T cells (↓), DC (↓)
TAM	TGF-α, TGF-β, IL-10, IL-6, Arg-1	Cancer cells (↑), Treg (↑), Th17 (↑) CD8^+^T cells (↓), NK cells (↓)
MDSC	Arg-1, NO, IL-6	Cancer cells (↑) CD8^+^T cells (↓), NK cells (↓)
DC	IL-23, TGF-β	Th17 (↑) CD8^+^T cells (↓)
Treg	TGF-β, IL-10	PSC (↑) CD8^+^T cells (↓), NK cells (↓)

↑, the function of cells is enhanced; ↓, the function of cells is inhibited. *aPSC* activated pancreatic stellate cells, *CAF* cancer-associated fibroblasts, *DC* dendritic cells, *MDSC* myeloid-derived suppressor cells, *NK cells* natural killer cells, *TAM* M2 tumor-associated macrophages, *Th17* helper T cells 17, *Treg* regulatory T cells

At the stage of tumorigenesis in pancreatic cancer, mutations of the KRAS gene cause ductal metaplasia of acinar cells, followed by pancreatic intraepithelial neoplasia (PanIN). During the progression from PanIN-I to PanIN-III, mutations in several key genes, including P16, TP53 and SMAD4 genes, ultimately lead to tumor formation. Theoretically, the immune microenvironment stimulated by tumor cells should fight against tumor cells and inhibit tumor formation. However, tumor cells escape immune system surveillance by low expression of major histocompatibility complex class I and overexpression of programmed death-ligand 1 (PD-L1), Fas, and Fas ligand. In addition, immune cells are reprogrammed to promote the formation of a “cold” tumor that is characterized by an absence of T cells within the tumor and at the tumor edges and failure of T cell priming [9].

Tumor-associated macrophages (TAM) are the most common infiltrating immune cells in the TIME and are categorized as either activated M1 or activated M2 macrophages. M1-TAM have antitumor functions, whereas M2-TAM contribute to tumor progression. Macrophages can be induced to M2-TAM by tumor cell–derived C-C motif chemokine ligand 2 (CCL-2), transforming growth factor beta (TGF-β), and intercellular adhesion molecule 1 (ICAM-1) and then enhance the malignant phenotype of tumor cells. Furthermore, M2-TAM secrete arginase 1 (Arg-1) and TGF-β that attenuate the function of CD8^+^  T cells and natural killer (NK) cells, which also cross talk with regulatory T cells (Treg) and type 1 helper T (Th1) cells by the secretion of interleukin-10 (IL-10) and TGF-β [10]. Cytokines in the TIME, such as granulocyte-macrophage colony stimulating factor (GM-CSF) and granulocyte colony stimulating factors (G-CSF), arrest the maturation of myeloid progenitor cells and induce differentiation to myeloid-derived immunosuppressor cells (MDSC) that exert immunosuppressive functions, including inhibition of T cells and NK cell proliferation and activation, induction of Treg expansion, and promotion of macrophage differentiation to M2-TAM [11–13].

The molecular mechanism of the immunosuppression of MDSC has been identified. MDSC are mainly classified into polymorphonuclear MDSC (PMN-MDSC, also called granulocytic-MDSC) and mononuclear-MDSC (M-MDSC) [14]. Both PMN-MDSC and M-MDSC express high amounts of Arg-1, which depletes L-arginine and results in T-cell inhibition [13]. PMN-MDSC and M-MDSC have distinct immunosuppressive mechanisms. PMN-MDSC produce high amounts of reactive oxygen species (ROS) that activate signal transducer and activator of transcription 3 (STAT3) [15]. M-MDSC express high levels of inducible nitric oxide synthase (iNOS), which activate the STAT1 signal and increase the production of nitric oxide (NO) [16]. MDSC exert immunosuppressive effects by interactions with immune cells. However, MDSC also promote tumor progression by directly contacting cancer cells. Panni et al. demonstrated that M-MDSC increased the population of cancer stem cells and promoted epithelial-mesenchymal transition when cocultured with pancreatic cancer cells through a STAT3-dependent mechanism [17].

The tumor stroma also plays a crucial role in the formation of the TIME. In the presence of cancer cells or during injury, pancreatic stellate cells (PSC) are activated, which inhibit the proliferation of T cells and NK cells and attenuate the cytotoxic functions, activate MDSC, and polarize macrophages by secreting cytokines [18, 19]. Furthermore, activated PSC secrete a large amount of extracellular matrix proteins that prevent therapeutic drugs into the tumor [18].

**Fig. 1:**
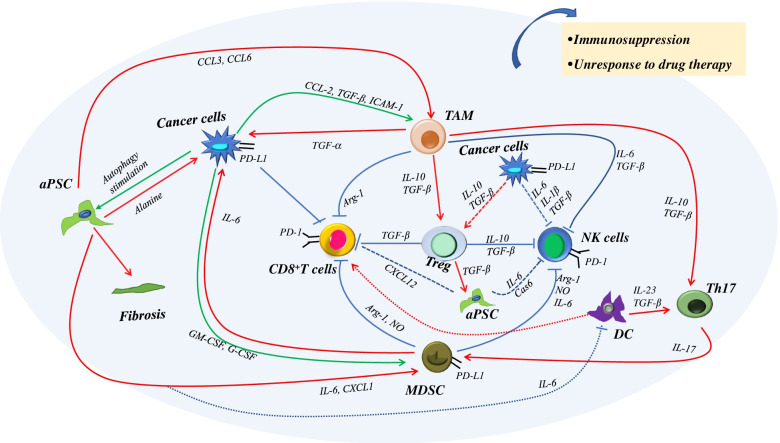
The tumor immune microenvironment in pancreatic cancer. Pancreatic cancer is focused as an example to describe the TIME. Tumor cells, immune cells, and stromal cells interacting with each other via cytokines constitute a “cold” tumor with abundant extracellular matrix. aPSC, activated pancreatic stellate cells; DC, dendritic cells; MDSC, myeloid-derived suppressor cells; NK cells, natural killer cells; TAM, M2 tumor-associated macrophages; Th17, helper T cells 17; Treg, regulatory T cells

In summary, tumor cells, immune cells, and stromal cells interacting with each other via cytokines constitute a “cold” tumor with abundant extracellular matrix, which is characterized by immunosuppression, and insensitivity to drug therapy (Fig. 1).

## Pyroptosis

**Fig. 2:**
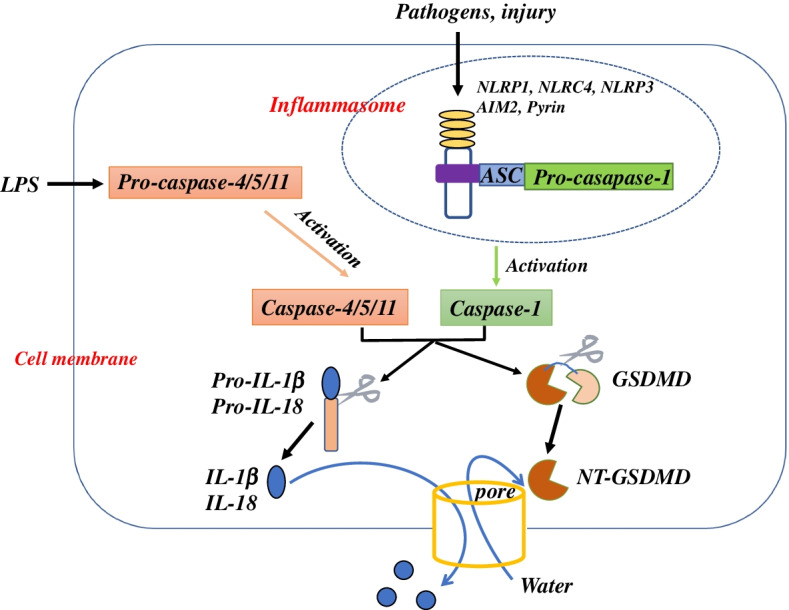
The pyroptosis pathways. In the canonical pathway, exogenous pathogens and endogenous damage are recognized by the intracellular sensor proteins, such as NLRP1, NLRC4, NLRP3, AIM2, and Pyrin, and then activate caspase-1. In the non-canonical pathway, intracellular LPS binds directly to caspase-4/5 in humans and caspase-11 in mice and results in activation of these caspases. Activated caspase-1/4/5/11 cleave GSDMD; the N-terminal domain is liberated, which forms pores on cell membrane and results in cytoplasmic swelling, membrane rupture, and the release of cytosolic contents. Activated caspases also cleave pro-inflammatory cytokines IL-1β and IL-18, leading to maturation of IL-1β and IL-18 that are released from the pores on the cell membrane and amplify the local or systemic inflammation

Pyroptosis is a gasdermin (GSDM)-mediated programmed cell death [20] that was first described in 1992 [21]. The GSDM family contains six members in humans (A, B, C, D, E, and DFNB59) and 10 members in mice (A1/2/3, B, C1/2/3/4, D, and DFNB59). All GSDM members in human except for DFNB59 contain two conserved domains (a C-terminal inhibitory domain and a N-terminal effector domain), which are cleaved and mediate pyroptosis [22]. Most research has focused on GSDMD and GSDME. GSDMD-meditated pyroptosis is induced by both the canonical inflammasome pathway and the non-canonical inflammasome pathway (Fig. 2). In the canonical pathway, exogenous pathogens and endogenous damage are recognized by intracellular sensor proteins, including the nucleotide-binding oligomerization domain (NOD)-like receptors (NLRP1, NLRC4 and NLRP3), absent in melanoma 2 (AIM2), and Pyrin. The signal recruits the inflammasome adaptor apoptosis-associated speck-like protein containing a caspase activation and recruitment domain (CARD) through Pyrin domains, bridging inflammasome sensors with pro-caspase-1 and resulting in activation of caspase-1 via self-cleavage [23]. In the non-canonical pathway, intracellular lipopolysaccharide (LPS) binds directly to caspase-4/5 in humans and caspase-11 in mice via the CARD domain, resulting in the activation of these caspases [24]. Activated caspase-1/4/5/11 cleave GSDMD, the N-terminal domain is liberated and forms pores on the cell membrane, resulting in cytoplasmic swelling, membrane rupture, and the release of cytosolic contents. Activated caspases also cleave the pro-inflammatory cytokines IL-1β and IL-18, leading to maturation of IL-1β and IL-18. These pro-inflammatory cytokines are released from the pores on cell membrane and amplify the local or systemic inflammatory effects [22].

GSDME-meditated pyroptosis is usually induced by drugs. Chemotherapeutic drugs, such as doxorubicin and cisplatin, activate caspase-3 and then cleave GSDME [25]. Other drugs such as metformin enhance B-cell lymphoma-2-associated X expression and cytochrome C release, subsequently mediating caspase-3/GSDME noncanonical pyroptosis [26]. Caspase-7 also cleaves GSDME at the same cleavage site as caspase-3 [27]. There are also reports about the cleavage of GSDMA, GSDMB and GSDMC, which are listed in Table 2.

**Table 2:** Induction and cleavage of gasdermin family

Gasdermin	Induced by	Cleaved by
**GSDMA**	Group A Streptococcus [28]	Streptococcal pyrogenic exotoxin B [28]
**GSDMB**	Viruses, allergens [29] IFN-γ [30]	Caspase-1 [31] Caspase-3/6/7 [29] Granzyme A [30]
**GSDMC**	TNF-α [32]	Caspase-8 [32]
**GSDMD**	Pathogens, injury, LPS	Caspase-1/4/5/11
**GSDME**	Doxorubicin, Cisplatin [25] Paclitaxel [33] Metformin [26]	Caspase-3/ 7[25] Granzyme B [34]

## Involvement of pyroptosis in the TIME

**Fig. 3:**
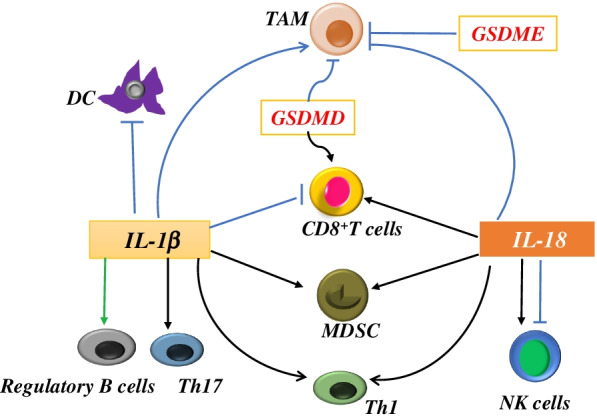
Involvement of pyroptosis in the TIME. The pyroptosis executor GSDM, and proinflammatory cytokines (IL-1β and IL-18) modulate the TIME by regulating immune cells

Evidence of the involvement of pyroptosis in TIME has mainly come from bioinformatic analyses, which demonstrates that several genes and non-coding RNAs related to pyroptosis are associated with tumor infiltrating immune cells in pan-cancers [35–37]. Here, we review the current understanding of the pyroptosis executor GSDM, inflammasome, and proinflammatory cytokines (IL-1β and IL-18) in modulating the TIME (Fig. 3).

**Fig. 4:**
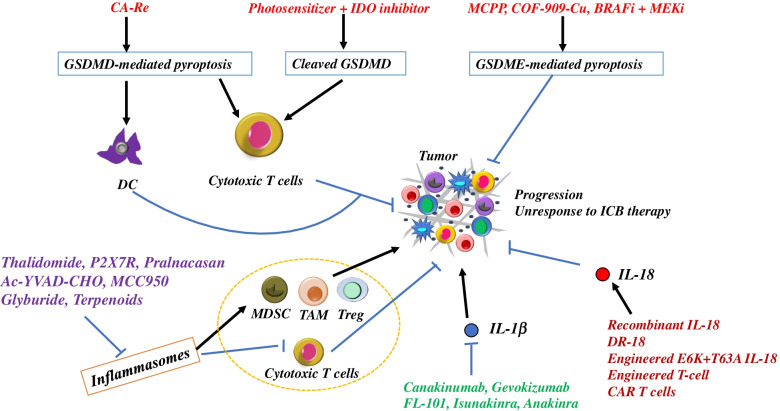
Remodeling the TIME by targeting pyroptosis. Several treatments targeting pyroptosis have been proposed or developed to overcome the immunosuppression microenvironment, including evocation of GSDMD/GDDME-mediated pyroptosis, prevention of inflammasome activation, antagonizing IL-1β, increase of IL-18. ICB, immune checkpoint blockade

### GSDM

Several publications have reported the association between GSDM and the TIME; however, most studies reported results that were obtained from the bioinformatic analyses. The associations of GSDMA and GSDMB with the TIME were just traced by bioinformatic analyses. However, some evidence from basic experimental studies have confirmed the association between GSDMC, GSDMD, GSDME and the TIME. Liu et al. developed an immunotherapy system on the basis of red blood cell membranes encapsulated *Listeria monocytogenes* (Lmo) with selective deletion of virulence factors (Lmo@RBC). Lmo@RBC-induced GSDMC-mediated cancer cell pyroptosis promoted the secretion of IL-6, TNF-α, and IFN-γ of bone marrow–derived DC and accumulation of DC and CD8^+^ T cells in tumor tissues [38]. Wang et al. [39] reported that membranous GSDMD expression in colorectal cancer positively correlated with CD68^+^ macrophages in the tumor center and CD8^+^ lymphocytes in the tumor invasive front. However, nuclear GSDMD was negatively related to CD68^+^ macrophages in the tumor invasive front and CD8^+^ lymphocytes in the tumor center. Cytoplasmic GSDMD was associated with more CD3^+^ lymphocytes both in the tumor center and tumor invasive front. The role of GSDME in tumor biology is unclear. Croes et al. indicated that GSDME played a role in tumorigenesis in intestinal cancer. However, neither the number of affected mice nor the multiplicity of proliferative lesions in both chemically induced and genetically modified intestinal cancer mouse models between the GSDME knockout and wild-type groups was different. The authors explained the contradictory phenomenon through the effects created by GSDME-mediated necrotic cell death on TIME due to the observation of inflammatory cell infiltration in the tumor [40]. Tan et al. [41] reported that macrophages were polarized to the M1 phenotype when cocultured with D2 dopamine receptor-expressing breast cancer cells, which then activated GSDME-mediated pyroptosis of breast cancer cells.

### Inflammasomes (Table 3)

#### NLRP3

Bioinformatic analyses identified NLRP3-inflammasome-related genes that were differentially expressed between normal and tumor samples in 15 cancer types and were significantly associated with survival of patients with hepatocellular carcinoma or skin cutaneous melanoma. The NLRP3 inflammasome score correlated with tumor-infiltrating lymphocytes and macrophages and was a stronger predictor for immune signatures compared with tumor mutation burden and glycolytic activity [42]. The NLRP3 inflammasome is involved in tumor progression by the recruitment of myeloid cells, such as MDSC and TAM [43]. The inhibition of inflammasomes suppressed TAM infiltration and increased CD4^+^ and CD8^+^ T cell infiltration and PD-L1 expression in tumors with high inflammasome signaling activity, amplifying the curative effect of PD-L1 blockade [44].

**Table 3:** Involvement of inflammasomes in TIME

Inflammasome	Role in regulating immune cells	Targeted therapy
NLRP3	TAM (↑), MDSC (↑), Treg (↑) CD8^+^ T cells (↓), NK cells (↓)	P2X7R antagonist Thalidomide Pralnacasan Ac-YVAD-CHO MCC950 Glyburide Terpenoids
NLRC4	TAM (↑), CD8^+^ T cells (↑) CD4^+^ T cells (↑), CD11b^+^ cells (↑)	
NLRP1	CD8^+^ T cells (↓), NK cells (↓) Th1 cells (↑)	
AIM2	Treg (↑) CD4^+^ effector T cells (↓), DC (↓)	

Note: ↑, the function of cells is enhanced; ↓, the function of cells is inhibited

The NLRP3 inflammasomes in tumor infiltrating immune cells and stromal cells are involved in tumor progression. Inflammasomes in macrophages are activated by the macrophage-colorectal cancer cell crosstalk, then increasing IL-1β secretion and promoting colorectal cell migration [45]. Cancer-derived exosomal tripartite motif-containing 59 activates the NLRP3 inflammasome in macrophages by ubiquitination and degradation of abhydrolase domain containing 5, which facilitated lung cancer growth and metastasis in vivo [46]. Inhibition of the NLRP3 inflammasome in macrophages suppressed the metastatic potential of melanoma cells [47]. Cancer-associated fibroblast–derived NLRP3 inflammasome and IL-1β facilitated tumor growth and metastasis by modulating the tumor microenvironment towards an immune suppressive milieu in breast cancer. The NLRP3 inflammasome increased the recruitment of CD11b^+^Ly6C^high^Ly6G^−^ myeloid cells into tumors, and IL-1β increased the recruitment of the granulocytic fraction (CD11b^+^Ly6G^+^Ly6C^low^) in the primary tumor and the monocytic fraction (CD11^b+^Ly6C^high^Ly6^G−^) in metastatic tumors [48].

Several factors activate the NLRP3 inflammasome and alter the TIME. Epstein-Barr virus latent membrane protein 1 promoted the expression of MDSC-related molecules and cytokines by activating the NLRP3 inflammasome, leading to tumor immunosuppression in nasopharyngeal carcinoma [49]. *Porphyromonas gingivalis* changed the TIME by selectively expanding myeloid-derived immune cells and inducing a proinflammatory microenvironment without the influence of CD4^+^ T cells, CD8^+^ T cells and NK cells in a colorectal cancer xenograft mouse model by activating the hematopoietic NLRP3 inflammasome [50]. High mobility group box 1 (HMGB1) increased the sensitivity of glioblastoma cells to temozolomide. Mechanistically, HMGB1 interacted with receptor for advanced glycation end products (RAGE), a major receptor for HMGB1 on TAM, and then activated the NLRP3 inflammasome by nuclear factor-kappaB (NF-κB) signaling, which polarized a M1-like macrophage phenotype by promoting the release of pro-inflammatory cytokines (TNF-α, interferon gamma (IFN-γ), IL-1β, IL-6, IL-8 and CCL2) [51]. The macrophage-specific deletion of glucose transporter 1 in pancreatic cancer mouse models inhibited tumor growth and NK cell infiltration by suppressing the pro-inflammatory NLRP3-IL1β axis [52].

#### NLRC4

NLRC4 is critical for cytokine production in TAM and necessary for the generation of IFN-γ-producing CD4^+^ and CD8^+^ T cells in an inflammasome-independent manner, suppressing tumor growth in a subcutaneous murine model of melanoma [53]. Obesity enhanced NLRC4 expression in tumor-infiltrating CD11b^+^ cells in breast tumors in mice, resulting in activation of IL-1β, and promotion of disease progression through adipocyte-mediated expression of vascular endothelial growth factor A and angiogenesis [54]. In mouse models for nonalcoholic fatty liver disease, fatty liver enhanced the expression of IL-1β and NLRC4 in metastatic tumors, which promoted TAM infiltration and M2 polarization and increased colorectal cancer liver metastasis [55].

#### NLRP1

Dipeptidyl peptidase (DPP) inhibitors induced NK and T cell infiltration, enhanced the efficacy of anti-PD1 antibody, and reduced tumor growth. Mechanistically, DPP inhibition activated the NLRP1 inflammasome, resulting in proinflammatory cytokine release and the Th1 cell response, further enhancing the CXCL9/10-CXCR3 axis [56]. However, DPP inhibitors also promoted the recruitment of tumor-infiltrating CD45, MPO, F4/80, CD4, Foxp3-positive cells, and MDSC and decreased CD8-positive lymphocytes in metastatic sites in mouse breast cancer models by ROS-NF-κB-dependent NLRP3 inflammasome activation [57]. The dual-role of DPP inhibitors in the TIME should be considered when the inhibitors are used in cancer treatment.

#### AIM2

Fukuda et al. reported that AIM2 promoted tumor growth with the increase of Treg proportion and decrease of CD4^+^ effector T cells and without changes in the numbers of CD8^+^ or CD4^+^ T cells and DC in the melanoma microenvironment in mouse models. Additionally, AIM2 inhibited the expression of IFN-β and increased IL-1β and IL-18. Tumor infiltrating DC were the major producers of IFN-β. The authors examined the expression of AIM2 in DC and found that tumor-infiltrating DC with AIM2-expression in human melanoma were increased and correlated with tumor progression. AIM2-deficient DC promoted tumor antigen–specific CD8^+^ T cell infiltration into the tumor via CXCL10. AIM2 was also required for the production of IL-1β and IL-18, which promoted Treg accumulation and tumor growth in vivo [58].

### IL-1β and IL-18

Both IL-1β and IL-18 lead to the infiltration of more immune cells and result in the generation and maintenance of an inflammatory microenvironment surrounding cancer cells [59], however, the biofunctions are diverse.

#### Il-1β

The function of IL-1β in the TIME is a double-edged sword in tumor progression. IL-1 signaling activates innate immune cells and drives polarization of CD4^+^ T cells towards Th1 and Th17 cells, which initiates an adaptive anti-tumor response [60]. The protective effects of IL-1β are observed in several tumors [60]. However, IL-1β also creates a tumor immunosuppressive microenvironment predominantly by TAM and MDSC, which promotes tumor development.

IL-1β enhances TAM expansion. Interferon-inducible protein 16 (IFI-16) is increased in pancreatic cancer, and elevated IFI-16 correlates to a poor prognosis. Overexpression of IFI-16 in pancreatic cancer cells induces the maturation, infiltration, and proliferation of TAM in the tumor microenvironment by activating inflammasomes and therefore increasing the release of IL-1β. Neutralization of IL-1β attenuated the effects of IFI-16 overexpression in pancreatic cancer cells on TAM [61]. Tumor cell–derived IL-1β is essential for the formation of the protumorigenic microenvironment in pancreatic cancer, which promotes the activation and secretory phenotype of quiescent PSC, increases stromal accumulation of TAM, MDSC, CD1d^hi^CD5^+^ regulatory B cells, and Th17 cells, and decreases intratumoral infiltration and activation of CD8^+^ cytotoxic T cells [62].

IL-1β also increases cell numbers and infiltration of MDSC in tumor. The levels of MDSC in mice harboring mammary carcinoma were positive association with IL-1β expression levels [63]. IL-1β upregulated cyclooxygenase-2 that encoded prostaglandins then mediate MDSC propagation [64]. IL-1β also activated STAT3 in glioma cells, which decreased the population of DC and increased the population of MDSC [65].

#### Il-18

Like IL-1β, IL-18 exhibits a dual-role in tumor development by regulating the TIME. The anti-tumor effects of IL-18 are partly ascribed to the production of IFN-γ in Th1 cells and NK cells. IL-18 synergized with IL-12 to promote IFN-γ production in Th1 cells [66], and Th1 cell–derived IFN-γ enhanced the cytolytic activity of CD8^+^ cells and NK cells. IL-18 also increased IFN-γ production and cytotoxic activity in NK cells without IL-12 [67] or combined with IL-12 or IL-2 [68]. Furthermore, IL-18 combined with IL-12 enhanced the cytotoxic activity of murine spleen-derived CD8^+^T cells, including inducing IFN-γ and granzyme production [69]. IL-18 in non-small cell lung cancer (NSCLC) tumor district, mostly produced by tumor cells, was associated with classical Th1 cell–associated cytokines (i.e., IFNγ, TNF-α, and IL-12) and contributed to the expansion of IL-18R^+^ CD8^+^ T cells expressing T-bet and IFN-γ within the tumor microenvironment [70]. Markowitz et al. reported that IL-18 played an inflammation-dependent tumor-suppressive effect mainly by promoting the differentiation, activity, and survival of tumor-infiltrating T cells in hepatocellular carcinoma [71].

IL-18 also promotes tumor progression by inducing an immunosuppressive microenvironment. Lim et al. demonstrated that IL-18 treatment significantly increased the percentage and the number of M-MDSC through the differentiation of bone marrow progenitor cells, which enhanced the suppression of T cell proliferation and IFN-γ production in vitro. The administration of IL-18 to mice bearing melanoma increased the accumulation of M-MDSC in the tumor microenvironment [72]. The multiple myeloma-niche-derived IL-18 drove the generation of both PMN-MDSC and M-MDSC, which suppressed CD8^+^T cell proliferation and IFN-γ production and then accelerated disease progression [73]. The tumor-promoting effects of IL-18 were also shown by IL-18 induction of PD-1 expression on NK cells in the TME, leading to a poor prognosis in nasopharyngeal carcinoma [74], and high expression of IL-18 in TAM promoted the migration and invasion of breast cancer cells [75].

Although IL-1β and IL-18 play crucial roles in creating the TIME, the regulatory role is not equivalent to the effect of pyroptosis in consideration of the source of IL-1β and IL-18 [60, 76]. The maturation and release of IL-1β and IL-18 depending on the inflammasome-caspase 1-GSDM signal is just one of the ways. Kiss et al. demonstrated that myeloid cells were the primary source of IL-1β in lung and breast tumors and released IL-1β independent of the inflammasome and GSDMD. Furthermore, the authors showed that IL-1β promoted systemic neutrophil expansion and enhanced the accumulation of T-cell-suppressive neutrophils in tumors. IL-1β deletion attenuated immune suppression in the TME, including inhibition of neutrophil recruitment and increase of cytotoxic CD8^+^T cells [77]. More evidence is needed to verify the effects of pyroptosis related to IL-1β and IL-18 in the TIME.

## Remodeling TIME by targeting pyroptosis: from bench to bedside (Fig. 4)

### Activation of GSDM-mediated pyroptosis

Both GSDMD and GSDME have been focused in the field of tumor-targeted therapy, and several small molecule agents or nano-drugs targeting GSDMD and GSDME have been developed. Su et al. [78] designed a carbonic anhydrase IX (CAIX)-anchored rhenium(I) photosensitizer (named CA-Re) that evokes GSDMD-mediated pyroptosis to effectively stimulate tumor immunogenicity. CA-Re enhanced the maturation and antigen-presenting ability of DC and fully activated the T cell–dependent adaptive immune response in vivo. A small molecule agent NBS-1MT with the photosensitizer and indoleamine 2,3-dioxygenase (IDO) inhibitor was synthesized that triggers pyroptosis by activating caspase-1 and then cleaving GSDMD. The activated pyroptosis enhanced the immune-related factor release and promoted the intratumoral infiltration of cytotoxic T lymphocytes and immunogenic cell death of tumor cells [79].

Xiao et al. [80] designed a smart tumor microenvironment ROS/glutathione (GSH) dual-responsive nano-prodrug (denoted as MCPP) with high paclitaxel and photosensitizer purpurin 18 (P18) loading that responded to high ROS/GSH in the tumor microenvironment and was shown to achieve optimal drug release in tumors. The nano-prodrug promoted the release of danger-associated molecular patterns via inducing GSDME-related tumor cell pyroptosis, which then initiated adaptive immunity, boosted the efficiency of the immune checkpoint blockade, achieved tumor regression, generated immunological memory, and prevented tumor recurrence. The engineering of a series of multienzyme-mimicking covalent organic frameworks (COFs), COF-909-Cu, induced GSDME-dependent pyroptosis and remodeled the tumor microenvironment, which activated durable anti-tumor immunity, enhanced the response of PD-1 inhibitor, and restrained tumor metastasis and recurrence [81]. Furthermore, activation of GSDM-mediated pyroptosis increased the effects of targeted therapy for tumors. The combinations of BRAF inhibitors and MEK inhibitors (BRAFi + MEKi) caused durable regression of melanoma by promoting cleavage of GSDME and the release of HMGB1. GSDME-deficient melanoma showed defective HMGB1 release, decreased tumor-associated T cells, and activated DC infiltrates in response to BRAFi + MEKi, and more frequent tumor regrowth was observed after drug removal [82].

### Inactivation of inflammasomes

Many agents and molecules have been developed to regulate inflammasome activity. However, few agents have been used in the clinic and several are still in clinical trials [83]. Thalidomide, a drug that inhibits the activation of caspase-1, showed anti-tumor effects in patients with advanced myeloma [83]. A phase II randomized clinical trial indicated that thalidomide combined with docetaxel resulted in the increased median survival in patients with metastatic androgen-independent prostate cancer [84]. However, thalidomide in combination with chemotherapy did not improve survival in patients with NSCLC and increased the risk of thrombotic events [85]. A P2X7R antagonist has been evaluated in a clinical trial. P2X7R promoted tumor growth in several solid tumors by activating the NLRP3 inflammasome. A phase I study was performed to assess the safety and tolerability of the P2X7R antagonist in the treatment of basal cell carcinoma; approximately 65% of patients showed a decrease of the lesion area and the most common adverse event was an allergic reaction at the treatment site [86]. Many agents, such as pralnacasan, Ac-YVAD-CHO, MCC950, glyburide, and terpenoids, are in the preclinical stage and have shown potential anti-tumor effects [83, 87]. Clinical trials are required to determine the potential clinical application of these agents.

Activation of the NLRP3 inflammasome remodeled the TIME and increased infiltration of MDSC, Tregs and TAM and decreased CD4^+^ and CD8^+^ T cells [88]. The genetic and pharmacologic inhibition of NLRP3 inhibited PMN-MDSC tumor infiltration and significantly improved the efficacy of anti-PD-1 antibody immunotherapy [89]. However, the combination of NLRP3 blockers with anti-PD-L1 treatment showed antagonistic effects in lymphoma. Lu et al. demonstrated that NLRP3 inflammasome blockade reduced the immunosuppressive cell population and PD-L1 expression and inhibited lymphoma growth. Anti-PD-L1 antibodies partly impaired the effect of MCC950 (an inhibitor of activation of NLRP3 inflammasomes) [90]. The possible reason was that activation of CD8^+^ T cells in response to PD-1 blockade induced a PD-L1/NLRP3 inflammasome signaling cascade that resulted in the recruitment of PMN-MDSC into the tumor, thus inhibited the resulting anti-tumor immune response [89]. This phenomenon indicates that a complex regulatory net with negative and positive feed-back loops is created in the TME, and inhibition of the NLRP3 inflammasome does not always only produce the desired positive effects. The application of NLRP3 inhibitors should be evaluated by preclinical studies.

### IL-1β and IL-18

#### Il-1β

A pre-clinical study indicated the anti-tumor effects of anti-IL-1β antibody and enhanced response of anti-PD-1 antibody [91]. Blocking IL-1R inhibited tumor growth and metastasis accompanied by inhibition of myeloid cell accumulation in human breast cancer models [43]. Several clinical trials for anti-IL-1β or IL-1R blockage in tumor treatment are ongoing.

The CANOPY program including four phase II or III randomized studies was designed to evaluate the effects of canakinumab (a IgG1k monoclonal anti-human antibody that selectively binds and neutralizes IL-1β) in the treatment of NSCLC [92]. The registered phase 1B study PanCAN-SR1 (NCT04581343, https://clinicaltrials.gov/) is recruiting to confirm the tolerable doses and acceptable safety profile of canakinumab and spartalizumab in combination with nab-paclitaxel and gemcitabine for treatment of patients with metastatic pancreatic cancer. Clinical trials for gevokizumab (an allosteric anti–IL-1β antibody) with standard care of anti-cancer therapies for metastatic colorectal, gastroesophageal and renal cancers, FL-101 (a monoclonal anti-IL-1β antibody) for surgically resectable NSCLC, isunakinra (a fusion protein-based inhibitor of the IL-1R containing domains from both IL-1β and IL-1Ra) for solid tumors, and anakinra (an IL-1 receptor antagonist) for hematological malignancies are ongoing.

#### Il-18

While preclinical studies and clinical trials have almost only focused on the anti-tumor effects of anti-IL-1β, other research has examined improving the levels of IL-18 in the treatment of tumors. The combination of recombinant IL-18 and immune checkpoint inhibitors led to synergistic inhibition of tumor growth in animal models of peritoneal dissemination of colon cancer [93]. The combination of IL-18 and IL-2 showed a synergistic anti-tumor effect in mice subcutaneously inoculated with a sarcoma [94]. However, recombinant IL-18 had limited activity as a single agent in patients with metastatic melanoma in clinical trials [95]. Several novel methods aiming to improve the anti-tumor effects of IL-18 have been developed.

Saetang et al. reported that engineered E6K + T63A IL-18, which has 16 times higher activity than native IL-18, increased the proportions of Th1 cells and cytotoxic T lymphocytes in mice harboring tumors and inhibited tumor growth [96]. Drakes and colleagues demonstrated that T-cell receptor (TCR)-modified T-cells with IL-18 expression created a superior proinflammatory TME, which improved overall survival of mice in the pmel-1 syngeneic tumor model [97]. Similarly, Kunert et al. engineered T cells with a melanoma-specific TCR and murine IL-18 under the control of a nuclear-factor of activated T-cell (NFAT)-sensitive promoter; the cells produced IL-18 and consequently enhanced levels of IFN-γ after exposure to antigen-positive tumor cells. Adoptive transfer of these cells to melanoma-bearing mice resulted in the accumulation of therapeutic CD8^+^T cells within tumors, reduction of tumor burden, and prolonged survival without side effects [98].

IL-18 binding protein (a high-affinity IL-18 decoy receptor) limited the anti-tumor activity of IL-18 in mice. Therefore, Zhou et al. engineered a “decoy-resistant” IL-18 (DR-18), which exerted potent anti-tumor effects in mouse tumor models compared with wild-type IL-18 by promoting the development of poly-functional effector CD8^+^T cells, decreasing the prevalence of exhausted CD8^+^ T cells, and expanding the pool of stem-like TCF1^+^ precursor CD8^+^ T cells. Additionally, DR-18 enhanced the activity and maturation of NK cells to effectively treat anti-PD-1-resistant tumors [99].

Chimeric antigen receptor (CAR) T cell therapy has emerged as an effective cancer treatment [100]. IL-18-secreting CAR T cells presented expansion and persistence and significantly increased long-term survival in mouse models for hematological and solid malignancies. These T cells modulate the TIME and enhance the anti-tumor immune response and might be a promising strategy to improve the clinical outcomes of adoptive T cell therapy [101].

Notably, both IL-1β and IL-18 exert dual effects in tumor progression. A protective effect of IL-1β in mouse models of chemically induced colon carcinoma was observed [102], whereas recombinant IL-18 promoted T-cell acute leukemia growth in vitro [103]. Therefore, more studies are needed to fully determine the role of IL-1β and IL-18 in tumors. The application of anti-IL-1β or recombinant IL-18 in the clinic should consider its dual role in tumors. Furthermore, several anti-tumor agents were shown to induce IL-1β mostly in a NLRP3 inflammasome–dependent manner [60]; therefore, the treatment of tumors with the combination of conventional agents and anti-IL-1β has been adapted. Voloshin et al. [104] demonstrated that paclitaxel chemotherapy induced IL-1β expression in macrophages in the TME, which promoted the invasive properties of cancer cells. Inhibition of IL-1β slightly delayed primary tumor regrowth following paclitaxel treatment but increased spontaneous metastases. The authors suggested that treatments using “add-on” drugs to conventional therapy should be investigated in tumor models consisting of primary tumors and their metastases [104].

## Conclusions and future perspectives

The TIME plays a key role in immunosuppression that results in tumorigenesis and tumor progression, as well as resistance to chemotherapy and immunotherapy. Pyroptosis exerts a crucial role in modulating the TIME. Several treatments targeting pyroptosis have been proposed or developed to overcome the immunosuppression microenvironment in tumor; however, most of these treatments are in preclinical studies. Although some agents have been used in the clinic, the outcomes are unsatisfactory.

In the field of drug development, researchers are also focusing on natural products and prescribed drugs. Several natural products associated with pyroptosis show anti-tumor effects, which are promising in developing strategies to improve the effects of chemotherapy and immunotherapy. Terpenoids are bioactive compounds widely distributed in nature and show a wide range of biological activities, such as antimicrobial, antiviral, anti-tumor, antioxidant, and cardio-protection effects [87]. Recently, these compounds were verified to inhibit the activation of the NLRP3 inflammasome in mouse models. Andrographolide, a natural diterpenoid from *Andrographis paniculate*, protected mice against azoxymethane/dextran sulfate sodium–induced colon carcinogenesis by inhibiting the NLRP3 inflammasome [105]. Triptolide, a compound isolated from the traditional Chinese herb *Tripterygium wilfordii Hook f,* eliminated head and neck cancer cells by inducing GSDME-mediated pyroptosis [106]. In addition, *Spatholobus suberectus* Dunn percolation extract exhibited anti-tumor efficacy by activating ROS-induced noncanonical inflammasome pyroptosis in triple-negative breast cancer [107]. Piperlongumine (PL) analogue L50377 suppressed the cell growth of non-small-cell lung cancer cells partly by activation of ROS-mediated pyroptosis [108].

Some prescribed drugs exhibit anti-tumor effects through regulating pyroptosis. Metformin, a widely used anti-diabetic medication, induces both GSDMD-mediated canonical pyroptosis [109] and caspase-3/GSDME-mediated noncanonical pyroptosis [26], which inhibited the proliferation of esophageal squamous cell carcinoma cells [109]. Disulfiram (DSF), an anti-alcoholic drug, was recently reported for the treatment of cancer and inflammatory disorders [110, 111]. However, the mechanism is not well understood. Hu et al. demonstrated that that DSF covalently modified Cys192 of GSDMD and inhibited pore formation of the plasma membrane and the process of pyroptosis [112]. Therefore, we speculated that one mechanism of the anti-tumor activity of DSF might be the inhibition of pyroptosis. Further studies are necessary to identify the potential clinical application of these compounds.

Notably, pyroptosis exhibits both anti-tumor and tumor promoting effects, which limits the application of agents targeting pyroptosis. The effects of agents that target pyroptosis should be well evaluated by rigorous preclinical studies and clinical trials. Furthermore, combined therapy with PD-1 antibody and NLRP3 inhibitor resulted in impaired anti-tumor effects compared with monotherapy by increasing recruitment of PMN-MDSC into the tumor, which highlights the complexity of the TIME. The combination of PD-1 antibody and targeted pyroptosis therapy in tumors might produce unexpected results. Nevertheless, deeper insights into the role of pyroptosis in modulating TIME expands our understanding of cancer and is beneficial to developing novel drugs for overcoming this tricky disease.

## Data Availability

Not applicable.

## Abbreviations

AIM2: absent in melanoma 2
Arg-1: arginase 1
CAR: chimeric antigen receptor
CARD: caspase activation and recruitment domain
CCL-2: C-C motif chemokine ligand 2
COF: covalent organic framework
DC: dendritic cell
DPP: dipeptidyl peptidase
DR-18: decoy-resistant IL-18
DSF: disulfiram
G-CSF: granulocyte colony stimulating factor
GM-CSF: granulocyte-macrophage colony stimulating factor
GSDM: gasdermin
GSH: glutathione
HMGB1: high mobility group box 1
ICAM-1: intercellular adhesion molecule 1
IFI-16: interferon-inducible protein 16
IFN-γ: interferon gamma
IL: interleukin
iNOS: inducible nitric oxide synthase
LPS: lipopolysaccharide
MDSC: myeloid-derived immunosuppressor cell
M-MDSC: mononuclear-MDSC
NF-κB: nuclear factor-kappaB
NK: natural killer
NLR: nucleotide-binding oligomerization domain (NOD)-like receptor
NOD: nucleotide-binding oligomerization domain
NSCLC: non-small cell lung cancer
PanIN: pancreatic intraepithelial neoplasia
PD-L1: programmed death-ligand 1
PMN-MDSC: polymorphonuclear MDSC
PSC: pancreatic stellate cell
ROS: reactive oxygen species
RAGE: receptor for advanced glycation end products
STAT3: signal transducer and activator of transcription 3
TAM: tumor-associated macrophage
TCR: T-cell receptor
TGF-β: transforming growth factor beta
Th1: type 1 helper T
TIME: tumor immune microenvironment
